# Tomographic and biomechanical refractive findings in ocular pediatric allergy

**DOI:** 10.1007/s10384-025-01252-w

**Published:** 2025-07-25

**Authors:** Wimolwan Tangpagasit, Suntaree Thitiwichienlert, Danaya Hongsuraphan

**Affiliations:** Department of Ophthalmology, Faculty of Medicine, Thammasat University, 95 Paholyothin Road, Khlong Laung, Pathum thani, 12120 Thailand

**Keywords:** Tomographic refractive findings, Biomechanical refractive findings, Ocular pediatric allergy, Corneal ectasia

## Abstract

**Purpose:**

To investigate corneal tomographic and biomechanical refractive findings in ocular pediatric allergy to identify high-risk eyes for ectasia.

**Study Design:**

A cross-sectional study

**Methods:**

Fifty-seven patients (5–18 years), diagnosed with allergic conjunctival disease (ACD) between August 2022 and September 2023, underwent comprehensive ocular examinations and clinical assessments. The Ocular Allergy Severity Score (OCAS Score) was calculated to assess the severity of signs and symptoms. Tomographic-based and biomechanical parameters, including Corvis Biomechanical Index (CBI), Tomographic and Biomechanical Index (TBI), Belin/Ambrósio Deviation (BAD-D), and KISA% index, with a cut-off value of 0.5, 0.79, 2.6 and 100, respectively, were employed to identify corneal ectasia.

**Results:**

Among 57 patients, 54.4% were diagnosed with vernal keratoconjunctivitis, 26.3% with seasonal allergic conjunctivitis, and 19.3% with atopic keratoconjunctivitis. The itching was the most common initial ocular symptom, followed by irritation and redness. The high-risk eyes for ectasia (based on CBI, TBI, BAD-D, and KISA%) were identified in 23.7%, 19.3%, 13.2%, and 7.9%, respectively. The ectatic group (determined by TBI) exhibited significantly higher mean curvature power (Km) (46.48±3.64 vs 43.10±1.31, p<0.001) and lower central corneal thickness (525.09±42.21 vs 561.20±30.91, p<0.001). However, there was no significant difference in the severity score of signs and symptoms between ectatic and non-ectatic groups. Isoametropic amblyopia was identified in 8.8% of patients, and anisometropic amblyopia was present in 1.8%.

**Conclusion:**

In ocular pediatric allergy, the risk of developing corneal ectasia is unrelated to the severity of signs and symptoms. Therefore, routine tomographic and biomechanical evaluations are recommended.

## Introduction

Ocular allergy, especially allergic conjunctival disease (ACD) is a prevalent ocular condition in pediatric medical practice. According to the largest epidemiologic study conducted by the International Study of Asthma and Allergies in Childhood (ISAAC) phase III, rhinoconjunctivitis prevalence in the Asia-Pacific region ranges between 3.6 and 17.7% in children aged 6–7 years and 4.8–21% in those aged 13–14 years [1]. Moreover, findings from The Global Asthma Network (GAN) phase I study report a 16.3% prevalence of rhinoconjunctivitis among 6,291 children in Bangkok, Thailand [2]. Notably, ocular allergy often coexists with systemic allergic conditions like asthma, allergic rhinitis, and atopic dermatitis, all of which exert considerable impact on daily life.

Pediatric ACD encompasses four primary classifications: seasonal allergic conjunctivitis (SAC), perennial allergic conjunctivitis (PAC), vernal keratoconjunctivitis (VKC), and atopic keratoconjunctivitis (AKC) [3]. SAC and PAC, which are more common, typically resolve without significantly affecting the ocular surface. However, VKC and AKC can induce severe inflammation on the ocular surface, potentially resulting in corneal scarring and subsequent vision impairment.

Keratoconus (KC) represents the most prevalent corneal ectatic disease, characterized by progressive corneal thinning and steepening, leading to a cone-shaped cornea, irregular astigmatism, and diminished visual acuity. Several identified risk factors for KC include a family history of keratoconus, eye rubbing, eczema, asthma, and allergies [4, 5]. The pathogenesis of ocular allergy and keratoconus involves an increase in protease enzymes, the inflammatory mediators released during allergic reactions and eye rubbing. Elevated protease activity coupled with decreased protease inhibitors leads to altered collagen configuration, contributing to keratoconus [6].

Given the characteristic corneal changes in KC involving progressive thinning and steepening, alterations in tomographic and biomechanical parameters serve as crucial tools for early KC detection. A study conducted by Wang et al. in 2021 identified statistically significant changes in various parameters among individuals with ACD compared to those without ACD. These changes include an increase in indices like index of surface variance (ISV), index of vertical asymmetry (IVA), keratoconus index (KI), index of height decentration (IHD), Belin/Ambrosio enhanced ectasia total deviation index (BAD-D), and Tomographic and Biomechanical Index (TBI), along with epithelial thinning. Furthermore, the study reveals a correlation between changes in epithelial thickness, both eyes rubbing frequency and allergic sign scores [7]. The keratoconus percentage index (KISA%) is calculated by multiplying the K value, the I-S value, the keratometric astigmatism index (AST), and the relative skewing of the steepest radial axes. A KISA% index greater than 100% has been found to be both sensitive and specific for diagnosing KC. The BAD-D assesses corneal shape to detect early ectasia, like KC. It is derived from Pentacam data, which evaluates anterior and posterior elevation and corneal thickness, with higher values indicating greater ectasia risk. The Corvis Biomechanical Index (CBI) is a score from the Corvis ST device, which measures corneal deformation under air pressure to assess biomechanical properties. It helps detect early keratoconus, with higher scores indicating a greater risk. TBI combines corneal tomography and biomechanical measurements (deformation under pressure) to evaluate corneal health and stability.

The authors hypothesized whether patients with pediatric ACD would have abnormal tomographic and biomechanical refractive findings that increase the risk of keratoconus or not. This current study aimed to investigate corneal tomographic and biomechanical refractive findings in pediatric ACD to identify high-risk eyes for ectasia and establish a correlation between these parameters and the severity of clinical signs and symptoms. The study primarily aimed to investigate corneal tomographic and biomechanical refractive findings in pediatric ocular allergy to establish a correlation between these findings and the OCAS Score of symptoms and signs. Additionally, the study seeks to identify eyes at high risk of developing ectasia based on these findings. The secondary outcome was to compare the intraocular pressure (IOP), mean corneal curvature power (Km), and central corneal thickness (CCT) between ectatic and non-ectatic groups, determined by using a TBI cut-off value of 0.79. Additionally, the study aims to screen patients within the study cohort for refractive amblyopia, including isoametropic and anisometropic amblyopia.

## Methods

The study design was a cross-sectional descriptive analysis. Fifty-seven patients diagnosed with ACD who attended the Ophthalmology Outpatient Clinic, Thammasat Hospital, Pathum thani, between August 2022 and September 2023 were included in the present study. The diagnoses of allergic conjunctivitis and clinical severity were made by a cornea specialist. This study was conducted with the approval of the Medical Ethics Committee of Thammasat University (MTU-EC-OP-1-122/65) from 8 August 2022 to 7 August 2023 in accordance with the tenets of the Declaration of Helsinki. Prior to participating in the study, the parents or guardians of all patients provided written informed consent.

The diagnosis of ACD was established based on clinical symptoms including itching, redness, photophobia, foreign body sensation, or increased mucous discharge, along with observable signs including conjunctival hyperemia, chemosis, tarsal conjunctival papillary reaction, or Horner-Trantas dots. SAC is defined as bilateral acute ACD that occurs seasonally. Since Thailand only has summer, rainy, and winter seasons, the spring and autumn seasons do not apply. PAC is defined as bilateral chronic, self-limiting ACD that can occur throughout the year. VKC is defined as bilateral chronic ACD that leads to a papillary response, mainly in the limbus or upper tarsus. AKC is defined as bilateral chronic ACD accompanied by dermatitis [8].

Patients aged between 5 to 18 years old were included in the study. Exclusion criteria encompassed: (1) patients with ocular media opacity that affected the cornea, lens, or vitreous humor, hindering ocular surface and refractive measurements, (2) patients diagnosed with amblyopia due to causes other than refractive error, (3) patients allergic to cyclopentolate, and (4) patients previously treated for allergies with controlled symptoms, defined as exacerbations occurring ≤ 4 days per week or < 4 weeks. A single examiner conducted a comprehensive ocular examination and clinical assessment of all participants.

### Demography and refractive appearance

The study collected demographic information comprising age, gender, underlying medical conditions, history of drug allergies, family history of allergies, exposure to pets, and a comprehensive history of ocular and systemic allergies. Subsequently, all patients underwent a complete ocular examination by slit-lamp biomicroscopy. A questionnaire was employed to evaluate the severity of ocular symptoms and signs. Symptoms including itching, tearing, photophobia, foreign body sensation, and irritation, as well as signs including eyelid swelling, lid margin inflammation, conjunctival injection and edema, eye discharge, tarsal conjunctival reaction, limbal involvement (such as Horner-Trantas dots), and corneal involvement (such as punctate epithelial erosion and Shield ulcer), were graded for severity on a scale ranging from 0 (indicating absence) to 4 (indicating seriousness). The evaluations were performed by a single clinician. The summation of the total scores obtained from these evaluations was represented as the Ocular Allergy Severity Score (OCAS Score) for symptoms and signs.

The initial visual acuity (Snellen chart) was assessed. Subsequently, cycloplegic refraction was conducted using 1% Cyclopentolate hydrochloride eyedrops [Batch No. N23G04, Mfg. Date 07/2023 Exp. Date 06/2025], administered as 1 drop every 5 minutes for 2 applications, followed by a 30-minute waiting period before performing retinoscopy. The Best Corrected Visual Acuity (BCVA) was determined during the second visit, wherein the participants were fitted with glasses based on the results obtained from the cycloplegic refraction assessment.

A diagnosis of refractive amblyopia was established based on a reduction in BCVA worse than 20/30 OU for bilateral amblyopia or an interocular difference of two or more lines, with the better eye maintaining normal visual acuity in unilateral amblyopia [9]. Amblyogenic refractive errors, which are severe refractive errors more likely to result in refractive amblyopia, were identified by the presence of 1.0–1.5 diopters (D) or more of hyperopia, 2.0 D or more of astigmatism, and 3.0–4.0 D or more of myopia in cases of anisometropic refractive error. In isoametropic refractive errors, involves the presence of 5.0–6.0 D or more of myopia, 4.0–5.0 D or more of hyperopia, or 2.0–3.0 D or more of astigmatism [10].

### Ocular Allergy Severity Score (OCAS Score) assessment

The OCAS Score was determined with modifications based on two previous studies [7, 11]. The severity of ACD symptoms was assessed based on a history of five symptoms: itchy eyes, watery eyes, light sensitivity, eye irritation, and eye pain. Each symptom was graded on a scale from 0 to 4 as follows: grade 0 = no symptoms, grade 1 = mild symptoms, grade 2 = symptoms present for half of the day, grade 3 = symptoms experienced for most of the day, and grade 4 = symptoms experienced throughout the entire day. The severity of ACD signs was assessed based on six signs: eyelid position, including eyelid skin texture and eyelid margin; conjunctival redness and edema; eyelid and tarsal conjunctiva inflammation; limbal involvement; and corneal involvement. Each sign was graded on a scale from 0 to 4, following the criteria established in the previous study [11].

### Biomechanical refractive assessment

The corneal tomographic parameters, including CCT at the corneal apex, Km, KISA% index, and BAD-D, were measured using Pentacam (Oculus, Inc.) version 1.27r11. The corneal biomechanical parameters, such as IOP, CCT, CBI, and TBI, were measured using Corvis ST (Oculus, Inc.) version 1.6r2223.

### Statistical analyses

The sample size was calculated based on Taro Yamane's sample size formula. The statistical analyses were conducted utilizing SPSS software version 23.0 (SPSS, Inc.). Clinical characteristics and outcome data are reported as percentages and mean values with standard deviation. Group means were compared through the use of an independent t-test and Mann-Whitney U-test. The threshold for statistical significance was established at p < 0.05 for all the tests performed.

## Results

Fifty-seven patients were included in the present study. Thirty-eight (66.7%) were boys and 19 (33.3%) were girls. VKC was the most prevalent type of ACD (54.4%), predominantly observed in boys, followed by SAC (26.3%) and AKC (19.3%). The majority of ACD patients (68.4%) also had underlying systemic allergies, with allergic rhinitis (AR) being the most common (64.9%), followed by asthma (8.8%) and atopic dermatitis (8.8%) (Table 1). Systemic allergy symptoms typically began around the age of 6.08±3.00 years, while ocular allergy symptoms manifested around the age of 9.88±3.83 years. The study revealed that systemic allergy symptoms typically preceded ocular allergy symptoms. The itching was the most frequent initial ocular symptom (59.6%), followed by irritation (38.6%) and redness (35.1%).

**Table 1:** Demographic and clinical characteristics of patients (Total 57 patients)

Characteristics	Number	Percentage (%)
Sex		
Female	19	33.3%
Male	38	66.7%
Age (years)		
6–12	31	54.4%
13 or more	26	45.6%
Mean age (years)	12.44 ± 2.99	
Diagnosis		
AKC	11	19.3%
SAC	15	26.3%
VKC	31	54.4%
Family history of allergy	21	36.8%
History of exposure to pet	24	42.1%
Drug allergy	3	5.3%
Systemic allergy	39	68.4%
Allergic rhinitis	37	64.9%
Asthma	5	8.8%
Atopic dermatitis	5	8.8%
Mean onset of systemic allergy (years)	6.08 ± 3.00	
Severity of systemic allergy		
Absence of symptoms	18	31.6%
Mild	31	54.4%
Moderate	5	8.8%
Severe	3	5.3%
Initial ocular symptoms		
Itching	34	59.6%
Irritation	22	38.6%
Redness	20	35.1%
Photophobia	4	7.0%
Tearing	4	7.0%
Eye discharge	5	8.8%
Blurred vision	5	8.8%
Excessive blinking	1	1.8%
Epiphora	1	1.8%
Lid edema	1	1.8%
Mean onset of ocular allergy (years)	9.88 ± 3.83	
OCAS Score		
Mean sign OCAS Score (from 0-28)	5.75 ± 6.11	
AKC	5.64 ± 7.52	
SAC	3.00 ± 2.39	
VKC	7.13 ± 6.51	
Mean symptom OCAS Score (from 0-20)	4.84 ± 3.37	
AKC	4.82 ± 2.18	
SAC	3.67 ± 2.61	
VKC	5.42 ± 3.93	

*OCAS* Score Ocular Allergy Severity Score, *AKC* atopic keratoconjunctivitis, *SAC* seasonal allergic conjunctivitis, *VKC* vernal keratoconjunctivitis

**Fig 1.**
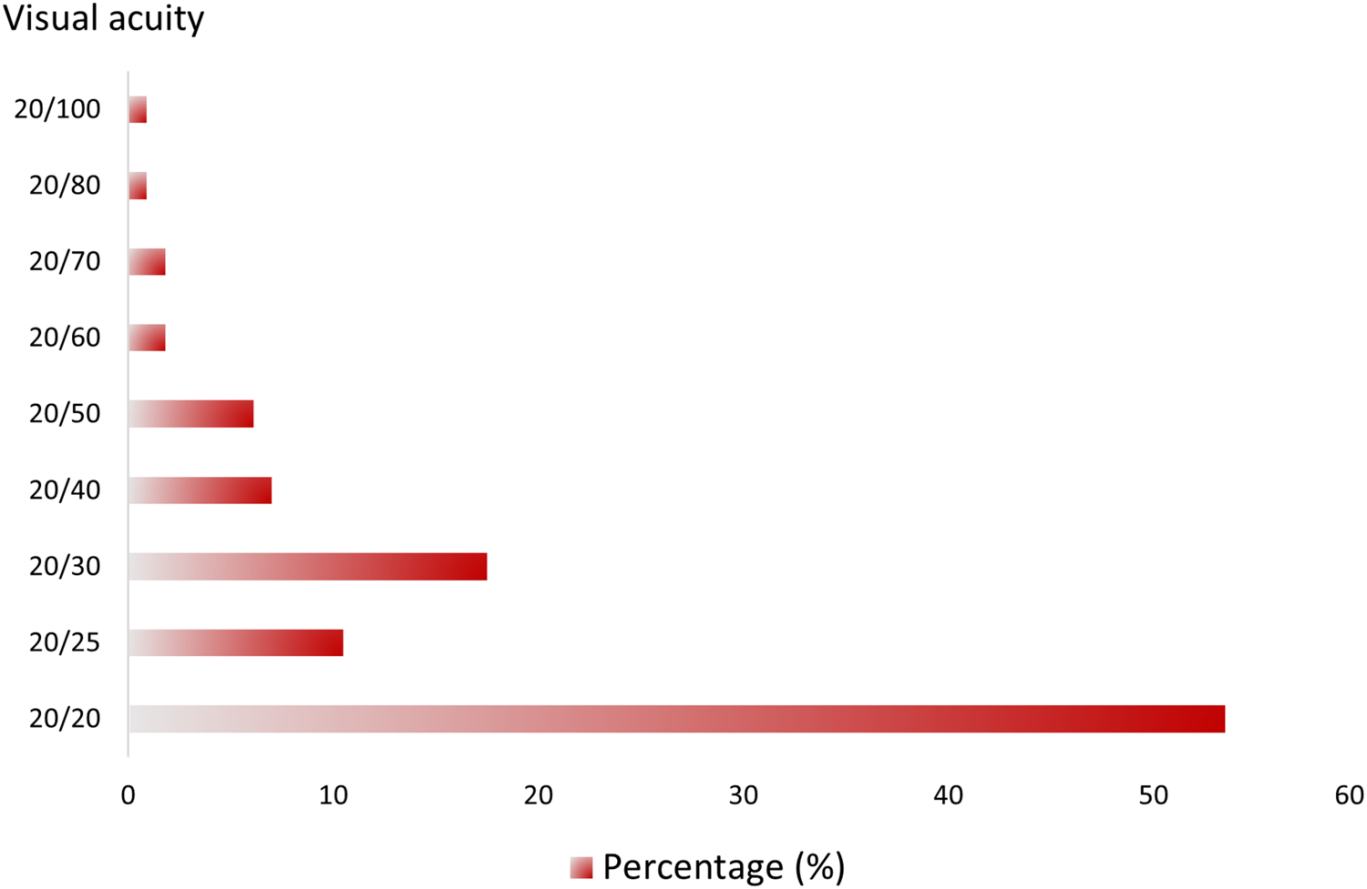
Best-corrected visual acuity (Total 114 eyes)

Regarding the grading of ACD symptoms and signs using the OCAS Score, the mean symptom OCAS Score was 4.84±3.37 (score range: 0-20), while for signs, it was 5.75±6.11 (score range: 0-28). Out of 114 affected eyes from 57 patients exhibiting ocular symptoms, a significant proportion maintained good vision, achieving BCVA of 20/20 (logMAR 0.0) in 53% of eyes. Additional ocular parameters are detailed in Figure 1 and Table 2.

**Table 2:** Ocular parameters (Total 114 eyes)

Parameters	Mean ± SD
LogMAR BCVA	0.12 ± 0.16
IOP (mmHg)	16.55 ± 3.81
Km (diopters)	43.75 ± 2.38
CCT (micrometers)	554.23 ± 36.13
CBI	0.29 ± 0.28
TBI	0.41 ± 0.33
BAD-D	1.90 ± 3.10
KISA%	80.39 ± 386.85

*CCT* central corneal thickness, *CBI* Corvis Biomechanical Index, *TBI* Tomographic and Biomechanical Index, *BAD-D *Belin/Ambrosio enhanced ectasia total deviation index, *KISA* K value, I-S value, keratometric astigmatism index

**Fig 2.**
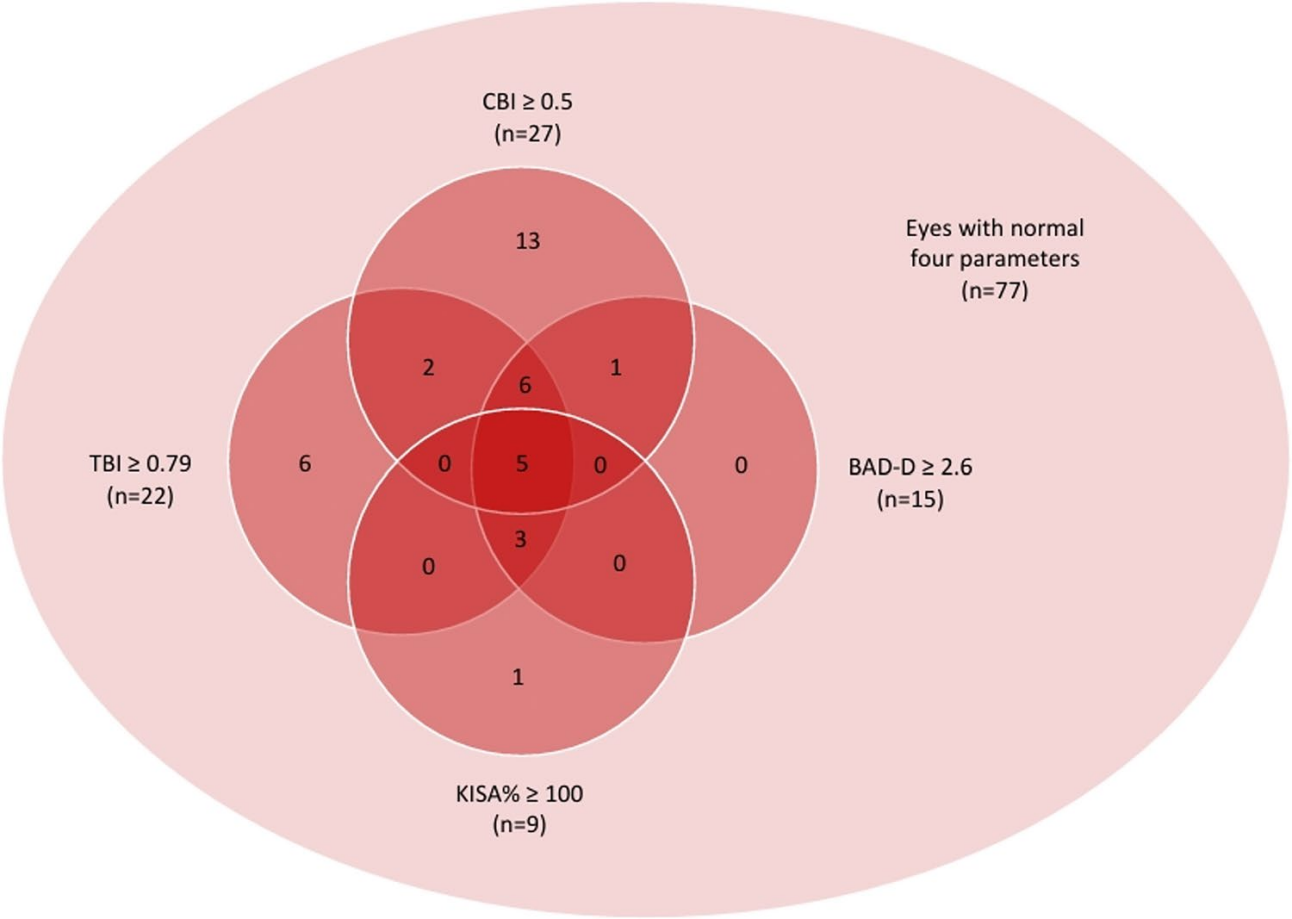
Ectatic eyes identified by four different parameters (Total 114 eyes)

Four parameters were employed to identify eyes at high risk for ectasia. Using CBI with a cutoff value of 0.5 (8), TBI with a cutoff value of 0.79 (9), BAD-D with a cutoff value of 2.6 (4), and KISA% index with a cutoff value of 100 (10), 27 eyes (23.7%), 22 eyes (19.3%), 15 eyes (13.2%), and 9 eyes (7.9%) were identified as a high risk for ectasia, respectively. Among the 114 eyes examined, 5 eyes exhibited abnormalities in all four parameters (Fig. 2). Among these 5 eyes, the mean symptom OCAS Score was 2.2±2.775, and the mean sign OCAS Score was 1.4±0.548. Thus, the likelihood of developing corneal ectasia did not appear to correlate with the severity of ocular signs and symptoms.

A comparison between the ectatic (using TBI with a cut-off value of 0.79) and non-ectatic groups is presented in Table 3. Regarding the type of ACD, we observed that the types of ACD,i.e. AKC, SAC, and VKC exhibited ectasia and non-ectasia equally, suggesting that there was no correlation between the corneal ectasia and the types of ACD (p=0.807). The OCAS Score of symptoms and signs did not significantly differ between these groups (p>0.05). However, the ectatic group exhibited significantly higher Km (46.48±3.64 vs. 43.10±1.31, p<0.001) and significantly lower CCT (525.09±42.21 vs. 561.20±30.91, p<0.001) based on the Mann-Whitney U-test and independent t-test. We used multivariate analysis to examine the risk of corneal ectasia (Table 4).

**Table 3:** Comparison between ectatic and non-ectatic group (Total 114 eyes)

Parameters	Ectatic group (22 eyes)	Non-ectatic group (92 eyes)	*p*-value
Mean symptom OCAS Score	4.41 ± 4.08	4.95 ± 3.18	0.236
Mean sign OCAS Score	4.50 ± 3.89	6.05 ± 6.48	0.208
IOP	14.80 ± 2.67	16.96 ± 3.94	0.021
Km	46.48 ± 3.64	43.10 ± 1.31	< 0.001
CCT	525.09 ± 42.21	561.20 ± 30.91	< 0.001
Type of allergic conjunctival disease			0.807
AKC (number, %) SAC (number, %)	4 (18.2%) 7 (31.8%)	18 (19.6%) 23 (25.0%)	
VKC (number, %)	11 (50.0%)	51 (55.4%)	

*OCAS* Ocular Allergy Severity Score, *IOP* intraocular pressure, *Km* mean corneal curvature power, *CCT* central corneal thickness, *AKC* atopic keratoconjunctivitis, *SAC* seasonal allergic conjunctivitis, *VKC* vernal keratoconjunctivitis

**Table 4:** Factors associated with ectatic group (Total 114 eyes)

	Univariate			Multivariate		
Factors	Crude OR	(95%CI)	*p*-value	Adj OR	(95%CI)	*p*-value
IOP	0.815	(0.689, 0.964)	0.017	0.717	(0.536, 0.959)	0.025
Km	2.614	(1.726, 3.960)	<0.001	3.186	(1.853, 5.477)	<0.001
CCT	0.966	(0.949, 0.983)	<0.001	0.985	(0.967, 1.004)	0.121

Univariate and multivariate logistic regression analysis

*IOP* intraocular pressure, *Km* mean corneal curvature power, *CCT* central corneal thickness

**Fig 3.**
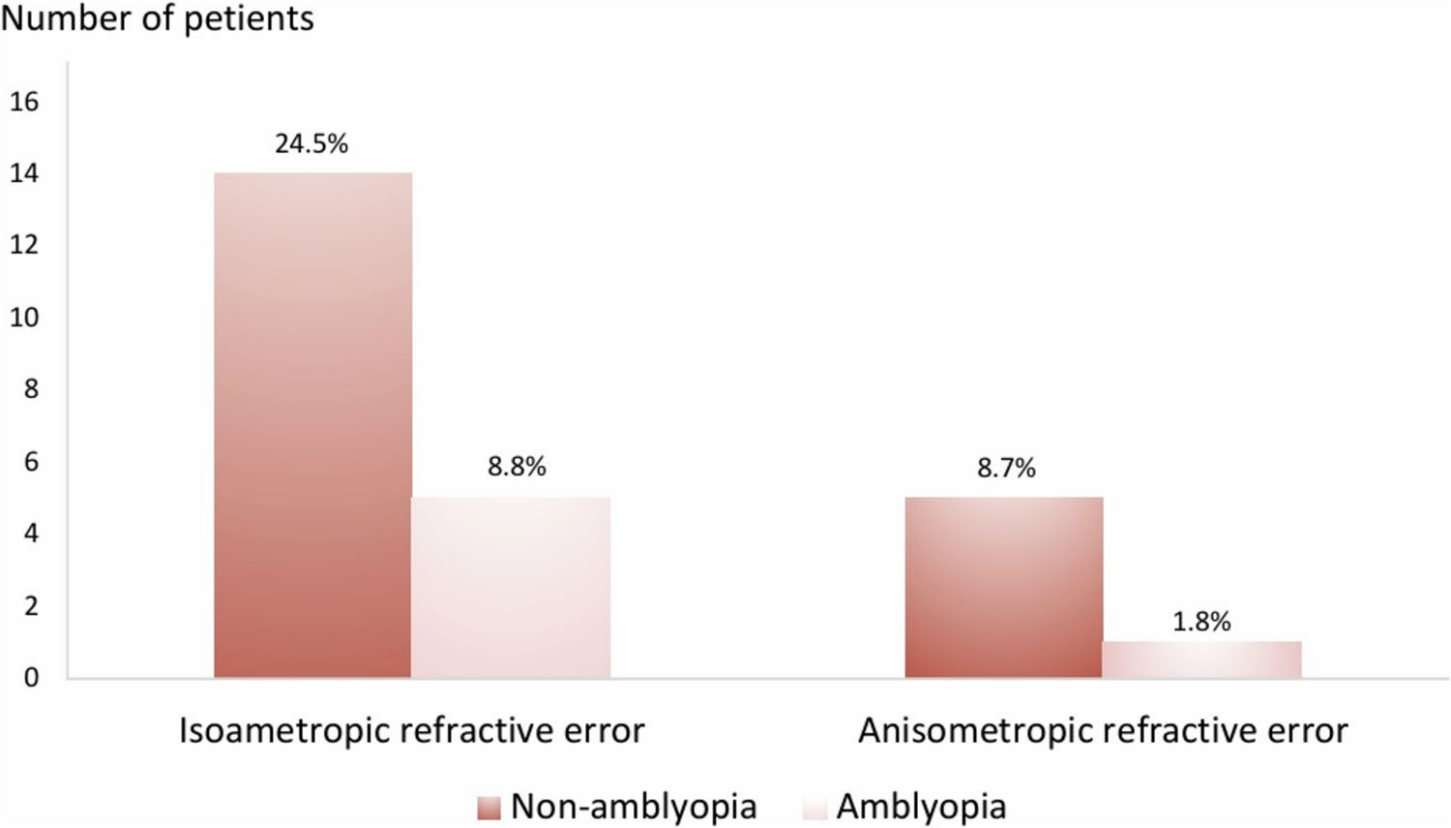
Eyes with amblyogenic refractive error (Total 25 eyes)

Ocular screening for amblyogenic refractive errors identified isoametropic refractive errors in 19 patients (33.3%), leading to amblyopia in 5 patients (8.8%), and anisometropic refractive errors in 6 patients (10.5%), resulting in amblyopia in 1 patient (1.8%) (Fig. 3). Corneal involvement was observed in 14 patients, leading to reduced BCVA in 6 patients. Subsequent treatment resulted in clinical improvement, restoring normal BCVA in 5 patients. However, 1 patient continued to exhibit reduced BCVA due to isoametropic amblyopia.

## Discussion

ACD stands as a prevalent condition in pediatric practice, evidenced by a reported 16.3% prevalence of rhinoconjunctivitis among children in Bangkok, Thailand [2]. Corneal involvement in ACD also includes superficial punctate keratitis (SPK), punctate epithelial erosions (PEE), shield ulcers, corneal neovascularization, and corneal scarring. SAC and PAC are more common types of ACD, whereas VKC and AKC are less common but more severe forms [12]. However, in our study, VKC emerged as the most prevalent type (54.4%), displaying a higher incidence among boys (66.7%), followed by SAC (26.3%) and AKC (19.3%). Notably, there were no reports of PAC in our study. A substantial majority of patients with ACD (68.4%) exhibited concomitant systemic allergies, with allergic rhinitis being the most prevalent (64.9%). Hence, it is imperative to investigate and manage systemic allergies in all patients with ACD.

There exists a heightened risk for ACD patients to develop KC. Pathogenesis is linked to proteases and various inflammatory mediators released during allergic responses and eye rubbing, which lead to collagen configuration alterations [5, 6]. KC is characterized by progressive corneal thinning and steepening, often identifiable through abnormal topographic parameters. Previous studies have identified corneal ectasia using tomographic and biomechanical parameters such as CBI, TBI, BAD-D, and the KISA% index [13–16]. The established cut-off values for assessing potential corneal ectasia are 0.5 for CBI, 0.79 for TBI, 2.6 for BAD-D, and 100 for the KISA% index [15]. Recent studies propose corneal biomechanical alterations and changes in epithelial thickness as early indicators of KC diagnosis. For instance, Vinciguerra et al. (2017) present a case series of subclinical KC patients, where one eye had normal topographic and tomographic findings, while the other had abnormalities through Corvis ST. Their findings indicate abnormal biomechanical results in both eyes, reinforcing the value of biomechanical analysis in diagnosing subclinical KC [17].

In our study, 22 eyes (19.3%) were identified as high risk for ectasia by TBI, 27 eyes (23.7%) by CBI, 15 eyes (13.2%) by BAD-D, and 9 eyes (7.9%) by the KISA% index. These findings suggest that changes in biomechanical parameters precede topographic and tomographic changes, advocating for the inclusion of biomechanical parameters, particularly CBI and TBI, in KC screening protocols.

Utilizing a TBI cutoff value of 0.79, established with 100% sensitivity and specificity [15], we categorized eyes with AC into ectatic and non-ectatic. Notably, the OCAS Score of symptoms and signs did not significantly differ between these groups (p>0.05). This indicates that the clinical severity of ACD did not correlate with the risk of corneal ectasia, differing from previous studies. For example, Wang et al. (2021) demonstrate associations between changes in epithelial thickness and allergic sign scores [7], while Mazotta et al. (2018) found a strong correlation between KC progression and allergy severity [18]. Based on our study, routine ectatic screening using tomographic and biomechanical evaluations is recommended for all pediatric ACD patients.

Our study unveiled a high prevalence of patients with amblyogenic refractive errors. A total of 25 patients (43.86%) were identified, with 19 patients (33.3%) having isoametropic refractive errors leading to amblyopia in 5 patients (8.8%), and 6 patients (10.5%) with anisometropic refractive errors resulting in amblyopia in 1 patient (1.8%). Beyond corneal ectatic screening, thorough evaluation for severe refractive errors is advised for all pediatric ACD patients. Timely correction of refractive errors is critical in pediatric amblyopia settings, as early intervention can prevent or ameliorate amblyopia, significantly impacting these patients' lifelong visual health.

A previous study in Thailand involving 164 children with allergic conjunctivitis found PAC to be the most common type (61.6%), followed by SAC (21.3%), VKC (12.2%), and AKC (4.9%) [8]. In contrast, our study found VKC to be the most prevalent (54.4%), followed by SAC (26.3%) and AKC (19.3%), with no cases of PAC. Therefore, our study may not fully represent the Thai population but highlights the importance of assessing the risk of corneal ectasia in children with certain types of ACD that involve keratoconjunctivitis.

Our study has some limitations. A tertiary center setting might potentially inflate the prevalence of more severe forms of ACD (VKC and AKC) compared to population-based prevalence figures. Consequently, this might lead to an overestimation of disease severity. Additionally, the limited sample size may restrict the generalizability of our findings to the broader population. Future studies with larger sample sizes are warranted to explore the intricate relationship between biomechanical changes and the severity of ACD. Moreover, our current investigation is a cross-sectional study. To better understand corneal biomechanical changes over time and to identify risk factors influencing progression, a prospective cohort study design would be essential.

In conclusion, our study findings suggest that the risk of developing corneal ectasia is not associated with the OCAS Score of signs and symptoms. As a result, we strongly recommend routine tomographic and biomechanical evaluations as a part of the standard assessment for these patients. Additionally, it is crucial to conduct comprehensive assessments for severe refractive errors in all cases because timely correction of refractive errors and early intervention play crucial roles in managing pediatric amblyopia.
