# Correction: Tomographic and biomechanical refractive findings in ocular pediatric allergy

**DOI:** 10.1007/s10384-026-01409-1

**Published:** 2026-08-14

**Authors:** Wimolwan Tangpagasit, Suntaree Thitiwichienlert, Danaya Hongsuraphan

**Affiliations:** Department of Ophthalmology, Faculty of Medicine, Thammasat University, 95 Paholyothin Road, Khlong Laung, Pathum thani, 12120 Thailand

**Correction: Japanese Journal of Ophthalmology** **https://doi.org/10.1007/s10384-025-01252-w**

In the Abstract section of this article, under Methods, in the sentence beginning, “Fifty-seven patients….” the month “July” should have been “August”.

The original publication has been corrected.

